# Olfactory Deficits and Mortality in Older Adults

**DOI:** 10.1001/jamaoto.2025.0174

**Published:** 2025-04-10

**Authors:** Robert Ruane, Oliver Lampert, Maria Larsson, Davide Liborio Vetrano, Erika J. Laukka, Ingrid Ekström

**Affiliations:** 1 Aging Research Center, Department of Neurobiology, Care Sciences and Society, Karolinska Institute and Stockholm University, Stockholm, Sweden; 2 Department of Psychology, Humboldt-Universität zu Berlin, Berlin, Germany; 3 Gösta Ekman Laboratories, Stockholm University, Stockholm, Sweden; 4 Stockholm Gerontology Research Center, Stockholm, Sweden

## Abstract

**Importance:**

Olfactory deficits are associated with higher mortality in older adults, but the mechanisms remain unclear. Further understanding this relationship could inform interventions to improve survival and quality of life for those with olfactory deficits.

**Objective:**

To investigate the association of olfactory deficits with all-cause and cause-specific mortality and to explore potential mediating factors.

**Design, Setting, and Participants:**

The Swedish National Study on Aging and Care in Kungsholmen (SNAC-K), is an ongoing population-based, longitudinal cohort study with baseline between 2001 and 2004. Eligible participants were residents of Kungsholmen, Stockholm, Sweden, and aged between 60 and 99 years from March 21, 2001, to August 30, 2004. Twelve-year follow-up was completed in February 2013. Data analysis took place between February 2024 and July 2024.

**Main Outcomes and Measures:**

Olfactory ability was tested with the 16-item Sniffin’ Sticks Odor Identification task. Mortality was determined through the Swedish National Cause of Death Register. Cox proportional hazards models examined the associations between olfaction and mortality over 6 years and 12 years. Competing hazard risks regression analyses assessed the olfactory-mortality association for specific death causes. Generalized structural equation models investigated mediators, including incident dementia, baseline chronic diseases, frailty, and malnutrition. The tested hypotheses were formulated after data collection.

**Results:**

Among 2524 participants (baseline mean [SD] age, 71.9 [10.0] years; 1545 [61.2%] female), 445 (17.6%) had died at 6 and 969 (38.4%) at 12 years of follow-up. Each additional incorrect answer on the odor identification test was associated with a 6% increased all-cause mortality risk at 6 years (hazard ratio [HR], 1.06 [95% CI, 1.03-1.08]) and 5% increased risk at 12 years (HR, 1.05 [95% CI, 1.03-1.08]) in multiadjusted models. In cause-specific models, the olfaction-mortality association had the greatest risk in relation to neurodegenerative death causes. Meaningful mediators for death at 6 years included dementia (23% of total association), frailty (11% of total association), and malnutrition (5% of total association). At 12 years, frailty remained a mediator (9% of total association).

**Conclusions and Relevance:**

The results of this cohort study underscore the importance of olfactory function as a mortality risk marker in older adults and highlight the evolving influence of neurodegeneration and frailty on this relationship. Further research is needed to assess the clinical utility of olfactory assessments in identifying individuals at risk of adverse health outcomes.

## Key Points

**Question:**

Is there an association between olfactory deficits and mortality in older adults, and what are the key mediating factors and cause-specific outcomes?

**Findings:**

In this cohort study of 2524 older adults, poorer olfactory function was associated with increased mortality, with the highest risk associated with neurodegenerative mortality; incident dementia, frailty, and malnutrition mediated the association at 6 years, with dementia being the most meaningful mediator (23%). Collectively, the mediators explained 39% of the association, while at 12 years, frailty remained the sole mediator, accounting for 9% of the association.

**Meaning:**

Olfactory deficits signal mortality risk, reflecting neurodegenerative and physiological decline, with mediators shifting over time.

## Teaser

This cohort study investigates the association of olfactory deficits with all-cause and cause-specific mortality in older adults and explores potential mediating factors.

## Introduction

Prospective studies consistently support olfactory impairment to be related to a heightened risk of mortality.^1,2,3,4,5,6,7,8^ A recent meta-analysis of 11 studies with a pooled cohort of 14 601 participants suggested that individuals with olfactory dysfunction have a 52% increased hazard of all-cause mortality compared to those with normal olfaction, with follow-up durations ranging from 3 to 10 years.^9^

Olfactory impairment may be linked with mortality through multiple mechanisms.^10^ Directly, it may impair hazard detection (eg, spoiled food, smoke, gas),^11^ though death due to such incidents is rare.^12^ Indirectly, it may reflect underlying conditions like neurodegeneration.^13^ Olfactory impairment is closely associated with Alzheimer^14^ and Parkinson diseases,^15,16^ with early neuropathological changes observed in brain regions supporting the olfactory system.^17^ However, research has produced conflicting findings on whether the olfaction-mortality link is primarily due to dementia present before death.^3,4,5,6,7,10^ Olfactory impairment may signal increased systemic inflammation, reduced neural plasticity, and diminished repair capacity, contributing to frailty and a decline in physical function and an increased vulnerability to illness and accidents.^18,19,20,21,22,23^ It may also indirectly elevate mortality risk through altered eating behaviors, leading to malnutrition.^24,25^ These pathways likely interact, positioning olfactory impairment as both a potential contributor to and marker of mortality risk.^10^

Olfactory deficits have also been linked to several chronic diseases, some of which may increase mortality risk,^26^ including hypertension,^27^ chronic kidney disease,^28^ cardiovascular diseases,^29,30,31^ obstructive lung diseases,^32^ diabetes,^33,34^ depression,^35,36^ and cancer.^37^ However, studies on the olfaction-mortality link for specific causes are limited. One study (participants aged 71-82 years) found associations with neurodegenerative and cardiovascular mortality but not cancer or respiratory deaths.^7^ Further research in broader age ranges is needed.^5,7^

This cohort study of adults aged 60 to 99 years examined the association between olfactory function and mortality at 6 years and 12 years after baseline. We investigated potential moderating pathways of age, sex, and genetic risk for Alzheimer disease (*APOE* ε4 allele) and explored the relationship between olfactory ability, cause-specific mortality, and mediating pathways linking olfaction to all-cause mortality.

## Methods

### Study Design, Setting, and Participants

Data came from the Swedish National Study on Aging and Care in Kungsholmen (SNAC-K), an ongoing population-based, longitudinal cohort study.^38^ Eligible participants were residents of Kungsholmen, Stockholm, Sweden, 60 years or older from March 21, 2001, to August 30, 2004. Participants younger than 78 years were followed up every 6 years, and participants 78 years and older were followed up every 3 years, with the 12-year follow-up completed in February 2013. Written informed consent was obtained from all participants or their proxies for cognitively impaired individuals. The study was approved by the Regional Ethical Review Board in Stockholm, Sweden, and adheres to the Strengthening the Reporting of Observational Studies in Epidemiology (STROBE) reporting guideline.

### Data Collection

We excluded individuals without olfactory assessments and those with prevalent dementia. Trained staff conducted face-to-face interviews and clinical examinations, with home visits for those unable to visit the research center. Age, sex, education, occupation, and smoking were assessed through interviews. Semantic memory was measured with SRB:1, a 30-item vocabulary task.^39^ DNA was extracted for *APOE* genotyping, categorizing participants as ε4 or non-ε4 carriers.

Comprehensive interviews, clinical examinations, laboratory tests, medication use, and data from the Swedish National Patient Register were used to define diseases according to the *International Statistical Classification of Diseases and Related Health Problems, Tenth Revision (ICD-10)* at baseline.^40^ Chronic diseases considered as mediators included cardiovascular disease (ischemic heart disease, heart failure, atrial fibrillation, and stroke), depression, and type 2 diabetes. Body mass index (BMI) was calculated as weight in kilograms divided by height in meters squared. A BMI lower than 18.5 was defined as having malnutrition, adhering to the definition by the European Society for Clinical Nutrition and Metabolism.^41^ Frailty was defined by the Fried phenotype, which includes slow walking, weak grip, weight loss, exhaustion, and low physical activity.^42,43^ Participants were categorized as robust (0 conditions), prefrail (1-2 conditions), or frail (3-5 conditions), as detailed elsewhere.^44,45^

Dementia diagnoses were made at baseline and follow-ups according to the *Diagnostic and Statistical Manual of Mental Disorders* (Fourth Edition, Text Revision) using a 3-step procedure^46,47^: a preliminary diagnosis by the examining physician, a second by a reviewing physician, and, in case of disagreement, a final diagnosis by an external neurologist. For those who died between 2 follow-up assessments, we complemented the diagnosis of dementia by (1) linking the SNAC-K database with the Swedish National Cause of Death Register and (2) reviewing the clinical and medical records of the participants who died between 2 follow-up assessments.

### Olfactory Assessment

Olfactory identification was assessed with the Sniffin’ Sticks Odor Identification task, a standardized tool with high test-retest reliability.^48,49,50,51^ Sixteen odors are presented using felt-tip pens for 5 seconds each. Participants first attempt to freely identify the odor; if unsuccessful, they select from 4 written options (1 correct, 3 incorrect). A correct response, whether free or cued, earns 1 point, with total scores ranging from 0 to 16. Scores were analyzed as a continuous variable to examine how performance nuances related to mortality. To aid interpretation, odor scores were reversed, with higher scores indicating worse olfactory performance. Secondary analyses categorized participants as anosmic (0-6), hyposmic (7-10), or normosmic (11-16) based on established norms.^48,49^

### Mortality Assessment

Cumulative all-cause mortality at 6 and 12 years was derived from the Swedish National Cause of Death Register between 2001 and 2016. Survival time was calculated by subtracting the date of death from the date of baseline assessment. Cause-specific mortality was classified using the following *ICD-10* codes^40^: G20, G301, G308, G309, F01, and F03 for neurodegenerative diseases; I00 to I99 for cardiovascular deaths; J00 to J99 for respiratory deaths; and C00 to D48 for cancer deaths. Analyses of other specific causes were precluded by small numbers.

### Statistical Analysis

The association between olfactory scores and mortality was assessed using Cox proportional hazards models. The fully adjusted model included the following covariates, all measured at baseline: age and sex, education and occupation, smoking, and semantic memory. These analyses were conducted separately for the 6- and 12-year follow-ups. Moderating pathways of age group at baseline (≥78 vs <78), sex, and *APOE* were assessed, testing the interaction between group membership, olfaction, and mortality. In case of a significant interaction, analyses were stratified to evaluate the olfaction-mortality link for each group. Competing risks regression analyses examined cause-specific mortality (neurodegenerative, respiratory, cardiovascular, cancer, and other causes). The Fine and Gray model^52^ was applied, categorizing participants as survivors, deceased from the specific cause, or deceased from competing causes. This method allowed participants with multiple reported causes of death to be included in each of the relevant analyses while controlling for competing risks through the subdistribution hazard approach.

We used generalized structural equation modeling (SEM) to assess mediation pathways. SEM is a multivariate statistical method that can estimate direct and indirect associations between variables within a single model. Calculations were based on standardized coefficients. Mediation pathways were calculated by multiplying SEM coefficients along mediation pathways and expressed as a percentage of the total association. Model fit was assessed using established metrics: root mean square error of approximation (RMSEA), comparative fit index (CFI), and Tucker-Lewis index (TLI). RMSEA of less than or equal to 0.06 and CFI or TLI greater than or equal to 0.95 indicate good fit. The model controlled for age, sex, education, occupation, smoking, and semantic memory. All covariates were linked to the primary predictor, outcome, and mediators. The model was estimated using maximum likelihood with missing values for incomplete data. Standardized estimates were reported to simplify interpretation. We used secondary SEMs to explore interactions between mediators of the olfaction-mortality link. Interaction terms were added to the full model described herein, with mediators selected based on results from the initial analyses. Data were analyzed with STATA, version 17 (StataCorp LLC). Statistical analysis was conducted between February 2024 and July 2024.

## Results

Among 4590 individuals eligible for the study, 3363 (73.3%) participated in the initial examination, with 2848 (84.7%) completing a neuropsychological assessment. A total of 264 individuals without olfactory assessments and 60 with prevalent dementia were excluded, leaving 2524 participants (baseline mean [SD] age, 71.9 [10.0] years; 1545 [61.2%] female). A complete profile of participant characteristics is shown in Table 1.

**Table 1.** Baseline Population Characteristics for the Total Sample

Characteristic	No. (%) (N = 2524)
Odor identification score, mean (SD)	11.57 (3.15)
Age, mean (SD), y	71.90 (10.00)
Education, mean (SD), y	12.19 (4.23)
Body mass index,^a^ mean (SD)	25.77 (4.06)
Semantic memory score, mean (SD)	22.79 (5.10)
Status at 6 y	
Alive	2079 (82.4)
Deceased	445 (17.6)
Status at 12 y	
Alive	1555 (61.6)
Deceased	969 (38.4)
Sex	
Female	1545 (61.2)
Male	979 (38.8)
Occupation	
Blue collar	513 (20.3)
White collar	2007 (79.5)
Missing	4 (0.2)
Smoking	
Never	1144 (45.3)
Former	997 (39.5)
Current	369 (14.6)
Missing	14 (0.6)
APOE ε4	
Carriers	695 (27.5)
Noncarriers	1682 (66.6)
Missing	147 (5.8)
Diabetes	
Present	232 (9.2)
Absent	2292 (90.8)
Depression	
Present	105 (4.2)
Absent	2419 (95.8)
Malnutrition	
Present	53 (2.1)
Absent	2471 (97.9)
Frailty status	
Robust	1095 (43.4)
Prefrail	1086 (43.0)
Frail	202 (8.0)
Missing	141 (5.6)
Cardiovascular disease	
Present	777 (30.8)
Absent	1747 (69.2)

^a^ Body mass index is calculated as weight in kilograms divided by height in meters squared.

After 6 years, 445 participants (17.6%) died, and after 12 years, 969 (38.4%) died. Over 6 years, 221 participants (8.8%) developed dementia, with 134 alive and 87 deceased by the end. Over 12 years, 369 participants (14.6%) developed dementia, with 243 alive and 126 deceased by the end. Mean baseline odor identification performance was 2.34 points (95% CI, 1.99-2.69) lower among those who died at 6 years and 2.33 points (95% CI, 2.08-2.58) lower among those who died at 12 years compared to survivors.

### Olfactory Function and All-Cause Mortality

In multiadjusted models, each incorrect answer on the odor identification test was associated with an approximately 6% increased mortality risk at 6 years and with a 5% increased risk at 12 years (hazard ratio [HR], 1.06 [95% CI, 1.03-1.09] and HR, 1.05 [95% CI, 1.03-1.08], respectively; Table 2). A score of 6 items less would correspond to a 42% increased risk of all-cause mortality at 6 years and a 34% increased risk at 12 years.

**Table 2.** Hazard Ratios (HRs) for All-Cause and Cause-Specific Mortality by Olfactory Identification Scores

Cause of death	Follow-up at 6 y		Follow-up at 12 y	
	No.	HR (95% CI)	No.	HR (95% CI)
All-cause models				
Unadjusted model	445	1.19 (1.16-1.22)	969	1.18 (1.16-1.20)
Multiadjusted model^a^	445	1.06 (1.03-1.09)	969	1.05 (1.03-1.08)
Cause-specific models^a^				
Neurodegenerative	35	1.28 (1.18-1.38)	126	1.19 (1.13-1.25)
Respiratory	84	1.11 (1.04-1.17)	199	1.06 (1.01-1.11)
Cardiovascular	259	1.07 (1.03-1.11)	592	1.04 (1.02-1.06)
Cancer	144	0.99 (0.94-1.04)	285	0.99 (0.95-1.03)
Other	40	0.95 (0.85-1.07)	84	1.06 (0.98-1.13)

Abbreviation: HR, hazard ratio.

^a^ Adjusted for age, sex, years of education, occupation, smoking, and semantic memory ability.

Analyses of odor identification cutoffs are illustrated in eFigures 1 and 2 in Supplement 1. They showed that anosmia increased mortality risk compared to normosmic individuals, with a 68% higher risk at 6 years (HR, 1.68 [95% CI, 1.28-2.22]) and a 67% higher risk at 12 years (HR, 1.67 [95% CI, 1.37-2.03]). Hyposmia was associated with a 23% increased mortality risk at 6 years (HR, 1.23 [95% CI, 0.98-1.55]) and a 22% increased risk at 12 years (HR, 1.22 [95% CI, 1.05-1.42]), showing similar magnitudes at both follow-ups.

### Interactions With Age, Sex, and APOE

There were no significant interactions for age group (≥78 vs <78 years) or sex at either 6 or 12 years. A moderation pathway of ε4-carrier status on the olfaction-mortality association at 6 years was observed (HR, 1.12 [95% CI, 1.05-1.20]), suggesting a 12% higher likelihood of mortality for every unit decrease in the odor identification score for ε4 carriers as compared to noncarriers. Stratified analysis similarly showed that for ε4 carriers, each item that was not correctly identified increased mortality risk by approximately 15% (HR, 1.15 [95% CI, 1.07-1.23]) as compared to 4% for noncarriers (HR, 1.04 [95% CI, 1.00-1.08]). At the 12-year follow-up, the interaction term yielded an HR of 1.04 (95% CI, 0.99-1.08), suggesting a minimal or no moderation pathway of ε4-carrier status on this association. Similarly, stratified analysis showed no differences in magnitude of associations between ε4 carriers (HR, 1.09 [95% CI, 1.04-1.13]) and noncarriers (HR, 1.04 [95% CI, 1.02-1.07]).

### Cause-Specific Mortality

Competing risks regressions showed that poorer olfactory identification was associated with varying magnitudes of risk for specific causes of death (Table 2). For neurodegenerative deaths, there were significant associations at 6 years and 12 years (HR, 1.28 [95% CI, 1.18-1.38] and HR, 1.19 [95% CI, 1.13-1.25], respectively). Extrapolating from these estimates, a score of 6 items less would correspond to an HR of approximately 4.40 (340% increased risk) at 6 years and 2.84 (184% increased risk) at 12 years. For respiratory and cardiovascular mortality, the estimated associations were smaller but notable. Respiratory mortality had HRs of 1.11 (95% CI, 1.04-1.17) at 6 years and 1.06 (95% CI, 1.02-1.11) at 12 years, slightly higher than all-cause mortality (HRs, 1.06 [95% CI, 1.03-1.09] and 1.05 [95% CI, 1.03-1.08], respectively). A score of 6 items less would increase the risk by 87% for respiratory and 50% for cardiovascular mortality at 6 years and by 42% and 34% at 12 years, respectively. No meaningful associations were found for cancer or other causes.

### Mediators of the Olfaction-Mortality Association

**Figure 1.**
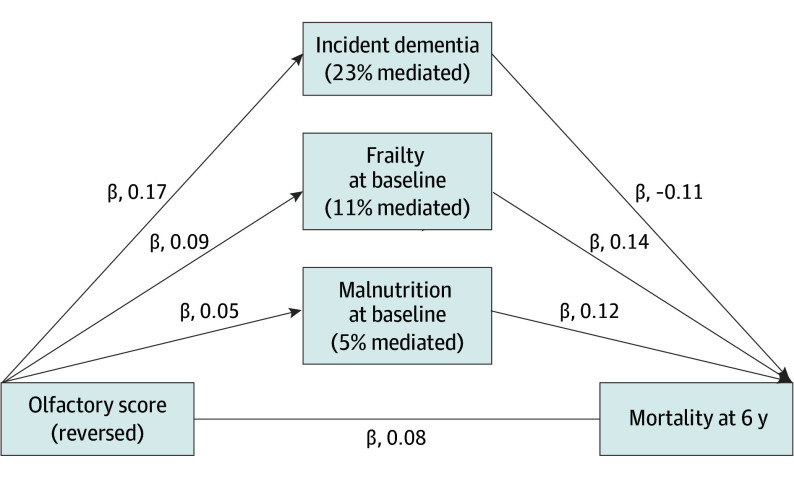
Mediators of the Olfaction-Mortality Association at 6 Years Path diagram of associations analyzed in the structural equation model, showing mediators with meaningful pathways linking olfaction to increased mortality at 6 years. Arrows indicate pathways with standardized coefficients representing the estimated associations. Percentages inside mediator boxes represent the proportion of the total estimated association mediated, calculated by multiplying the coefficients for the reversed olfactory score–mediator and mediator-mortality associations, dividing the mediated association by the total association, and multiplying by 100.

The results of SEM models are summarized in Table 3. They showed that all theorized mediators were associated with 6-year mortality, but odor deficits were only associated with incident dementia, malnutrition, and frailty (Figure 1). Partial mediation pathways were found for incident dementia, accounting for 23% of the total association, frailty accounting for 11%, and malnutrition accounting for 5%. Together, these mediators explained 39% of the olfaction-mortality association at 6 years. The model showed good fit with no significant differences in the likelihood ratio tests: χ^2^ = 0.015, compared to the saturated model, and a significant difference from the baseline model (χ^2^_76_ = 2823.659; *P* < .001). The RMSEA was 0 (90% CI, 0-0.02; *P* > .99), indicating good fit. CFI and TLI were 1.00 and 1.03, respectively.

**Table 3.** Coefficients and 95% CIs for Mediating Pathways Between Olfaction and Mortality From Structural Equation Modeling^a^

Pathway	*β (*95% CI)	
	Follow-up at 6 y	Follow-up at 12 y
Olfactory score to mortality	0.10 (0.06 to 0.14)	0.08 (0.05 to 0.12)
Olfactory score to mediators		
Incident dementia	0.17 (0.13 to 0.21)	0.17 (0.13 to 0.21)
Malnutrition	0.05 (0.00 to 0.09)	0.05 (0 to 0.09)
Frailty	0.09 (0.04 to 0.13)	0.08 (0.04 to 0.12)
Cardiovascular disease	0.01 (−0.03 to 0.06)	0.01 (−0.03 to 0.06)
Depression	0.04 (−0.01 to 0.08)	0.04 (−0.01 to 0.08)
Diabetes	0.03 (−0.02 to 0.07)	0.03 (−0.02 to 0.07)
Mortality to mediators		
Incident dementia	−0.11 (−0.15 to −0.07)	0.03 (−0.06 to 0.06)
Malnutrition	0.12 (0.09 to 0.15)	−0.03 (0 to 0.06)
Frailty	0.14 (0.10 to 0.18)	0.09 (0.05 to 0.12)
Cardiovascular disease	0.10 (0.07 to 0.14)	0.12 (0.08 to 0.15)
Depression	0.05 (0.02 to 0.08)	0.03 (0 to 0.06)
Diabetes	0.05 (0.01 to 0.08)	0.04 (0.01 to 0.07)
Olfactory score to mediator interactions^b^		
Incident dementia × frailty	0.14 (0.10 to 0.18)	0.15 (0.11 to 0.19)
Incident dementia × malnutrition	0.05 (0.01 to 0.09)	0.05 (0.01 to 0.09)
Frailty × malnutrition	0.02 (−0.02 to 0.07)	0.01 (−0.04 to 0.05)
Mediator interactions to mortality^b^		
Incident dementia × frailty	−0.14 (−0.28 to −0.00)	0 (−0.10 to 0.10)
Incident dementia × malnutrition	−0.02 (−0.13 to −0.08)	−0.10 (−0.20 to −0.08)
Frailty × malnutrition	0.07 (0.02 to 0.12)	0.01 (−0.04 to 0.05)

^a^ All structural equation models were adjusted for age, sex, years of education, occupation, smoking, and semantic memory ability.

^b^ Mediator interactions: interaction pathways between mediating variables in the model. Interactions with mediators (eg, incident dementia × frailty) represent combined associations with mortality risk.

**Figure 2.**
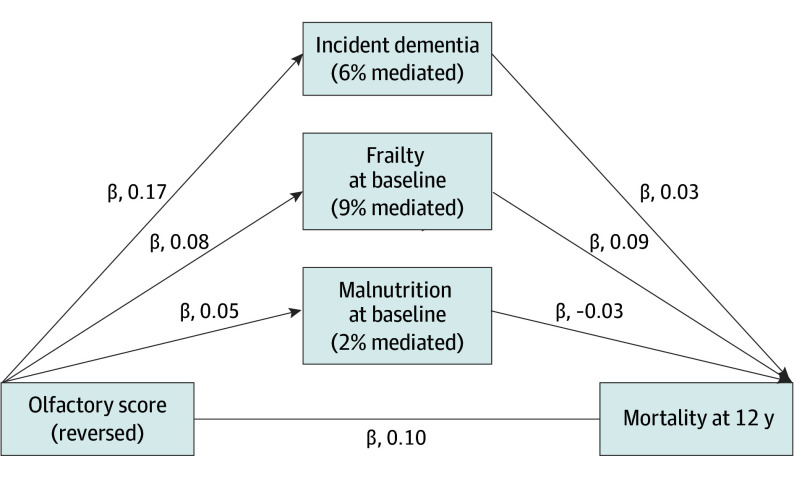
Mediators of the Olfaction-Mortality Association at 12 Years Path diagram of the estimated associations analyzed in the structural equation model, showing mediators with meaningful pathways linking olfaction to increased mortality at 12 years. Arrows indicate pathways with standardized coefficients representing the estimated associations. Percentages inside mediator boxes represent the proportion of the total estimated association mediated, calculated by multiplying the coefficients for the reversed olfactory score–mediator and mediator-mortality associations, dividing the mediated association by the total association, and multiplying by 100.

At 12 years, malnutrition, frailty, cardiovascular disease, and diabetes were associated with mortality, and olfaction was associated with malnutrition and frailty. Further examination of the indirect associations found that frailty remained a partial mediator, accounting for 9% of the total association (Figure 2). CFI and TLI values were 1.00 and 1.02, respectively.

At 6 years, the combined association of frailty and dementia with mortality was associated with lower olfactory scores and decreased mortality risk compared to those with neither or only one of these conditions. Similar but smaller associations were found for the interaction between dementia and malnutrition at both follow-ups. The frailty-malnutrition interaction slightly increased mortality risk at 6 years but showed no association with olfactory deficits or mortality at 12 years.

## Discussion

This longitudinal cohort study supports the existence of an olfaction-mortality link,^1,2,3,4,5,6,7,8,9^ with each additional incorrect odor identification increasing mortality risk by approximately 5%, and key mediators of the association included incident dementia, frailty, and malnutrition. Regarding cause-specific mortality, olfactory function had the highest likelihood of neurodegenerative deaths.

We did not detect sex differences in the olfaction-mortality link, consistent with findings from a large US cohort study.^7^ This may reflect olfactory deficits acting as a universal marker of health or offsetting sex-specific health trajectories, such as women’s higher resilience or men’s earlier mortality.^53^ Associations with mortality may be stronger in younger populations in whom olfactory impairment is rarer and more indicative of specific pathology, while its high prevalence in older adults may dilute this association.^5,7^ However, we found no moderating pathway of age, possibly due to the relatively healthier participants in SNAC-K, even among the oldest cohort.^54^

The HRs of olfaction increasing the risk of mortality were moderate for deaths related to neurodegenerative causes, slightly higher for respiratory-related deaths than for all-cause mortality, similar to all-cause mortality for cardiovascular-related deaths, and negligible for cancer and other remaining causes. In cancer, it has been proposed that olfaction is predominantly affected by chemotherapeutic or irradiation treatment and not by the disease itself.^55^ Our findings align with previous research on cause-specific mortality,^7^ though that study found no link between olfactory dysfunction and respiratory deaths. This discrepancy may reflect differences in age ranges, as the participants in our study included younger individuals. A recent study highlights concurrent olfactory decline and frailty in older adults with lung diseases.^56^

Our analysis of theorized mediators supports the involvement of incident dementia, as well as baseline frailty and malnutrition. Depression, diabetes, and cardiovascular disease did not emerge as mediators. Dementia explained 23% of the olfaction-mortality association at 6 years, closely aligning with a previous study.^7^ However, this association was absent at 12 years, corroborating other results.^4,5,6^ The role of dementia in the olfaction-mortality link may vary with follow-up length, potentially explaining discrepancies across studies. Relatedly, we found higher risks of mortality in individuals with worse olfactory function who were carrying the ε4 allele at 6 years but not at 12 years. By the 12-year mark, the cohort may include survivors who are either more resilient, have slower disease progression, or are more distant from the initial olfactory assessment, which could attenuate the impact of incident dementia on mortality observed in the earlier period. *APOE* ε4 carriers may be prone to earlier onset^57^ and more rapid progression of dementia,^58,59^ rendering the olfaction-mortality link more apparent at an earlier follow-up.

Frailty accounted for 11% of the total association at 6 years and remained the only meaningful mediator at 12 years, accounting for 8% of the total association. These findings build on prior research linking olfactory function to physical decline and emphasize recent calls to prioritize olfactory health in older adults with frailty.^18,60^ At 6 years, the interaction between frailty and dementia amplified olfactory deficits but lowered mortality risk. Most likely, this suggests overlapping pathways, such as genetic alterations, immune system dysfunction, neuroinflammation, or vascular burden.^61,62^ Malnutrition explained 5% of the association between olfaction and mortality at 6 years, consistent with previous findings,^7^ though this pathway was not replicated at 12 years. The interaction between malnutrition and dementia slightly amplified olfactory deficits while lowering mortality risk at both follow-ups, but effect sizes were small. We found no meaningful interaction between frailty and malnutrition in relation to olfactory function. Future research should explore dietary patterns to better understand the influence of nutrition on the olfaction-mortality link. Favorable diets have been associated with reduced frailty^63^ and improved cognitive function^64^ in individuals with olfactory impairment.

### Strengths and Limitations

Comprehensive data enabled analysis of cause-specific mortality and key mediators of the olfaction-mortality link. However, the homogeneous Stockholm-based cohort limits generalizability to populations with diverse exposures to environmental toxins affecting olfactory function and mortality.^65,66,67^ Our mediation models assessed baseline measures of chronic diseases, frailty, and malnutrition but considered the longitudinal incidence of dementia. This approach, guided by theoretical considerations, differs from classical mediation analysis, typically assessing all mediators at the same time point. In neurodegeneration, the olfaction-mortality link is likely driven by pathology preceding the clinical diagnosis of dementia,^68^ whereas trajectories of physical and sensory frailty may overlap, occurring proximal to the onset of olfactory impairment.^19^ Focusing solely on baseline mediators would overlook the progression of dementia, while relying exclusively on incidence data might inflate the association with dementia and understate the roles of frailty and chronic diseases, as these typically manifest earlier.^69,70^ Future research focusing on longitudinal changes in assessed mediators would complement our approach.

This study focuses on olfactory identification, integrating peripheral and higher-order processing.^71^ Although olfactory threshold and discrimination could not be assessed, these measures are associated with frailty and adverse health outcomes.^72,73^ Future research should examine more closely how specific olfactory subdomains relate to mortality.

## Conclusions

In this cohort study, we replicated the relationship between olfactory function and all-cause mortality, with the highest likelihood of death observed for neurodegenerative causes and followed by respiratory causes of death. Our results further underscore the evolving influence of frailty and neurodegeneration on the olfaction-mortality relationship. Overall, our findings reinforce olfactory deficits as a marker, rather than a direct contributor, to health outcomes linked to increased mortality.

## Notes

Supplement 1. eFigure 1. Survival curves over 6 years according to odor identification score cutoffs eFigure 2. Survival curves over 12 years according to odor identification score cutoffs

Supplement 2. Data sharing statement
