# Supplementary material for: Olfactory Deficits and Mortality in Older Adults
Source: JAMA Otolaryngol Head Neck Surg. 2025 Apr 10;151(6):558–66. doi: 10.1001/jamaoto.2025.0174 (PMC11986833; doi:10.1001/jamaoto.2025.0174)
Supplement: Supplement 1. — eFigure 1. Survival curves over 6 years according to odor identification score cutoffs eFigure 2. Survival curves over 12 years according to odor identification score cutoffs [file jamaotolaryngolheadnecksurg-e250174-s001.pdf]

Supplemental Online Content

Ruane R, Lampert O, Larsson M, Vetrano DL, Laukka EJ, Ekström I. Olfactory Deficits and Mortality in Older Adults. *JAMA Otolaryngol Head Neck Surg.* Published online April 10, 2025. doi:10.1001/jamaoto.2025.0174

eFigure 1. Survival curves over 6 years according to odor identification score cutoffs

eFigure 2. Survival curves over 12 years according to odor identification score cutoffs

This supplemental material has been provided by the authors to give readers additional information about their work.

© 2025 Ruane R et al. *JAMA Otolaryngology–Head & Neck Surgery*.

eFigure 1. Survival curves over 6 years according to odor identification score cutoffs

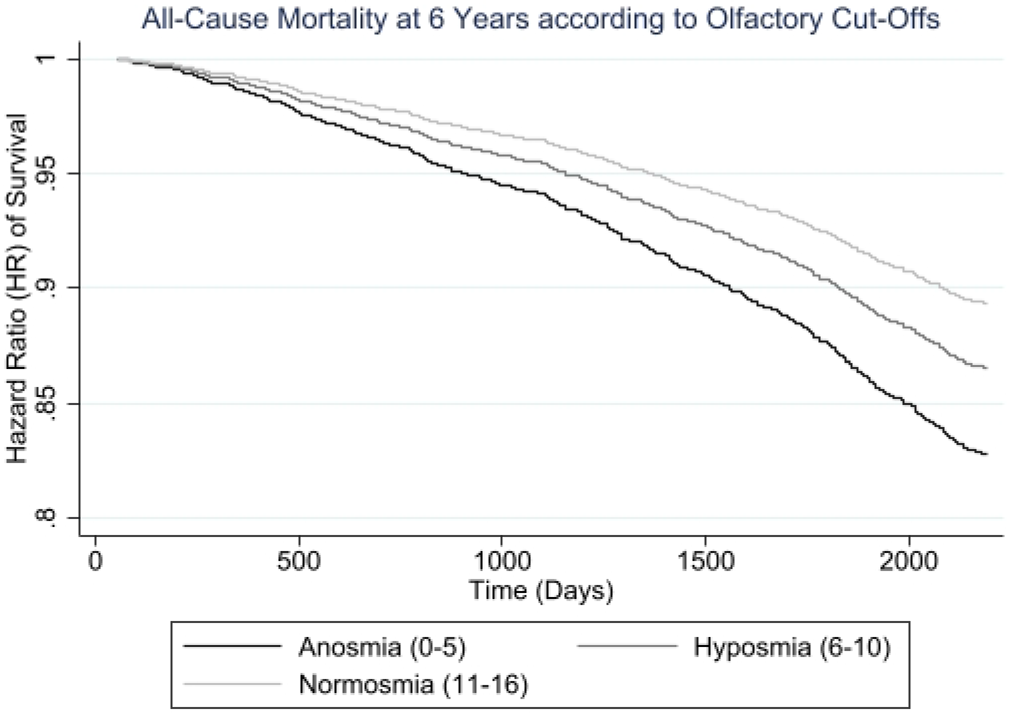

Legend: "Anosmia" (0-5, black line), "Hyposmia" (6-10, gray line), and "Normosmia" (11-16, light gray line). The Y-axis shows Hazard Ratio (HR) of survival and the X-axis shows follow-up time measured in days.

© 2025 Ruane R et al. *JAMA Otolaryngology–Head & Neck Surgery*.

eFigure 2. Survival curves over 12 years according to odor identification score cutoffs

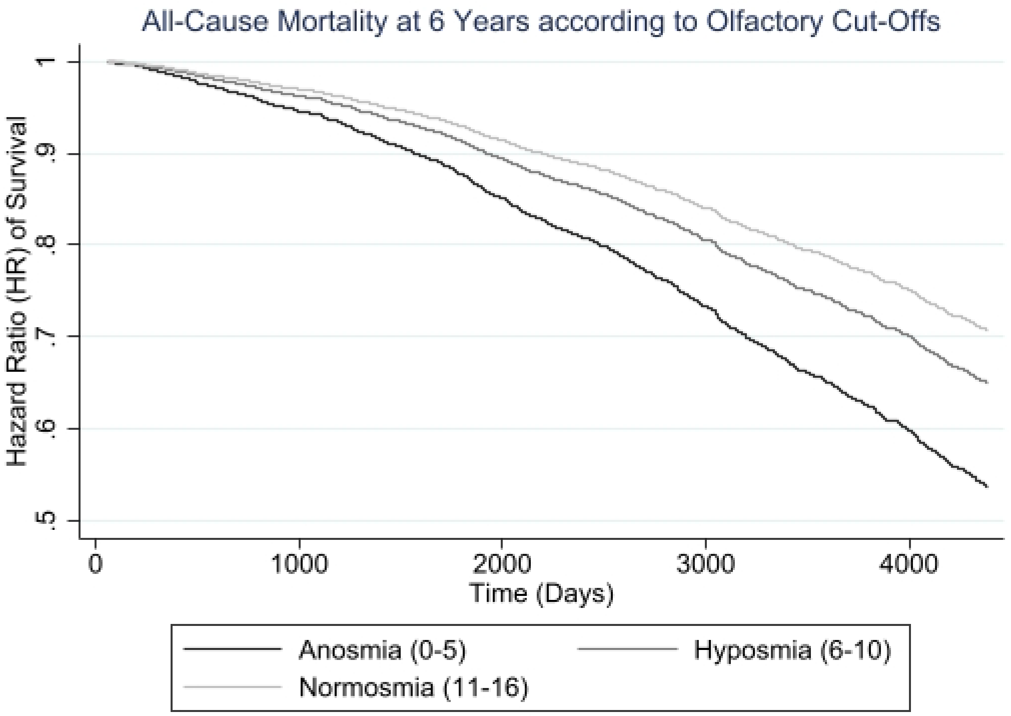

Legend: "Anosmia" (0-5, black line), "Hyposmia" (6-10, gray line), and "Normosmia" (11-16, light gray line). The Y-axis shows Hazard Ratio (HR) of survival and the X-axis shows follow-up time measured in days.

© 2025 Ruane R et al. *JAMA Otolaryngology–Head & Neck Surgery*.
