# Supplementary material for: Olfactory Deficits and Mortality in Older Adults
Source: JAMA Otolaryngol Head Neck Surg. 2025 Apr 10;151(6):558–66. doi: 10.1001/jamaoto.2025.0174 (PMC11986833; doi:10.1001/jamaoto.2025.0174)
Supplement: Supplement 2. — Data sharing statement [file jamaotolaryngolheadnecksurg-e250174-s002.pdf]

Data Sharing Statement

Ruane. Olfactory Deficits and Mortality in Older Adults. *JAMA Otolaryngol Head Neck Surg*.
Published April 10, 2025. doi:10.1001/jamaoto.2025.0174

Data
Data available: No

Additional Information
Explanation for why data not available: Deidentified data from the population-based Swedish National Study on Aging and Care in Kungsholmen (SNAC-K) are available upon reasonable request (https://www.snac-k.se/for-researchers/applicatio n-form/).
